# Co-producing childbirth knowledge: a qualitative study of birth stories in antenatal sessions

**DOI:** 10.1186/s12884-019-2605-z

**Published:** 2019-11-26

**Authors:** Leah de Quattro

**Affiliations:** Centre for the History of Science, Technology and Medicine, University of Manchester, Simon Building 2.54, Brunswick Street, Manchester, M13 9PL UK

**Keywords:** Childbirth, Antenatal, Education, Knowledge, Birth stories, Storytelling, Group-led, Participant-led

## Abstract

**Background:**

Birth stories surround pregnant women. Existing research on childbirth knowledge suggests that personal accounts from family and friends play a foundational role upon which other information builds. However, among the handful of studies that specifically address the educational role of birth stories, stories appeared to have little impact on knowledge creation. This paper engages with this discussion by exploring how birth stories contributed to the co-construction of birth knowledge within the specific context of antenatal sessions. Findings draw from the pilot study of a project which seeks to understand how women use collective approaches to co-produce birth knowledge.

**Method:**

Research data drew from participant observation of group-led Homebirth sessions (25 participants) and teacher-led National Childbirth Trust classes (18 participants). The researcher analysed transcripts using template analysis, a form of thematic content analysis, with principles from feminist ethnography and narrative analysis.

**Results:**

Storytelling proved central to mother-to-mother antenatal group practices, providing not only information, but also a means for understanding. This educational work took place through various mechanisms: Stories (re)shaped expectations, shared practical techniques, navigated different truth claims and approaches to knowledge, and helped build supportive communities of parents. These findings emerged more prominently in group-led sessions compared to teacher-led sessions.

**Conclusion:**

Compared to teacher-led norms, storytelling and other collective approaches to antenatal education provide additional resources to childbearing women. As dialogic, complex and flexible learning tools, stories offer uniquely diverse, credible and supportive messages. The next phase of this project will further investigate these findings, explore informal collective practices, and seek to evaluate the impact of collective knowledge on childbirth experiences.

## Background

Birth knowledge research suggests that stories form a crucial part of the network of birth knowledge that women build and use in preparation for childbirth [1–5]. However, most birth story research analyses storytelling only for its impact on the teller (e.g., [6–8]) or for information on maternity experiences (e.g., [9–13]). Meanwhile, mainstream British antenatal education has remained teacher-led, despite multiple scholars – and NICE guidelines – promoting group-led practices [14–17]. This paper engages with literature on childbirth knowledge and storytelling by focusing on the work of birth stories told within the specific context of antenatal sessions. Through participant observation, this research explored how stories worked to co-produce birth knowledge.

### Birth knowledge: situated and collective

Initial research on birth knowledge reports that women rely on a wide, shifting and individualised range of sources and processes for credible birth knowledge. Via large surveys in America, Australia and Britain, women have identified the usefulness of sources such as midwives, doctors, classes, the internet, books, television, family and friends [18–20]. Scholars report a range of types of childbirth knowledge, including formal expertise, lay knowledge, intuition, experience and more [21, 22]. Studies also suggest that birth knowledge develops continuously and dynamically, for example as parents interpreted messages delivered by educators within the context of personal circumstances, experiences and pre-existing ideas [4, 23, 24].

These findings resonate with the flexible approach to learning advocated by feminist epistemologists, in which dialogue, multiplicity, fluidity and an awareness of context work to construct credible knowledge [25–27]. Donna Haraway summarises this strategy as “situated knowledge” [28], recognising that authentic truth is also partial, and located firmly within bodies and communities.

### The educational role of birth stories

Birth stories appear in many studies regarding birth knowledge or childbirth generally. Still, most studies examined stories for their impact on the teller (e.g., [6–8]) or for information on maternity experiences (e.g., [9–13]). Only a few studies, all based on in-depth interviews, investigated the effects of birth stories on listeners [1, 29, 30].

A key finding in two studies recognised that women’s preconceived notions played a significant role in silencing or amplifying the stories they heard [29, 30]. Munro noted that “congruence between the nature of the message and the participants’ own views” usually determined credibility ([29], p., 376). For Kay et al., participants tended to dismiss stories that fell outside expectations as untrue, and repeat ‘horror stories’ that reinforced norms around medically-managed childbirth [30]. In fact, researchers concluded that “stories did not create meaningful knowledge” ([30], p., 10). Given the prominence of stories in the literature on birth knowledge, this scepticism about the educational impact of birth stories benefits from further exploration.

### Narrative ambush

General theories on storytelling help to contextualise birth story research. Frank proposed the notion of ‘narrative habitus,’ the personal, ever-changing repertoire of stories that affect interpretation of further stories [31]. This inner library may subconsciously determine whether someone experiences a story as interesting or boring, credible or unbelievable, enjoyable or unpleasant. Frank described narrative habitus as “the intuitive, usually tacit sense that some story is *for us* or not for us” ([31], p., 53).

However, Frank also suggested that some stories retain the capacity for ‘narrative ambush,’ surprising listeners, challenging expectations and reshaping the narrative habitus: “Vital, breathing stories can break through the filters and grids” ([31], p., 59). Extending Frank’s metaphor, an effective ambush may require not only powerful stories, but other conditions – storytellers, listeners, setting and other contexts – conducive to vulnerability.

Reflection on existing birth story research, with narrative ambush in mind, offers insights into the function and limits of birth stories as educational tools. For example, Munro’s participants formed a minority of birthing women who chose elective Caesarean section in the face of biological and institutional norms [29]. While Munro notes flexibility and diversity within this stance [29], perhaps it engendered a certain resistance to challenge or narrative ambush.

In Kay and colleagues’ study, researchers used a broad category of the ‘modern birth story’ as any social or media representation of childbirth [30]. This definition might have masked variations between different formats and contexts for stories. Indeed, a review of literature on the impact of media portrayals of childbirth suggested a narrow set of messages – birth as a dangerous, medical event [32]. Meanwhile, other birth story scholars have recognised that ‘everyday stories’ – oft-rehearsed, public versions, told quickly or casually – fundamentally differ from in-depth personal accounts which go beyond standard tropes [10, 11, 33, 34]. While variable interpretations of birth stories render effects unpredictable, this flexibility also provides a powerful, responsive resource for listeners.

### The unpredictable work of birth stories

Existing research on birth knowledge and experience imply other potentials for birth stories as educational tools. Scholars have identified birth stories as context-specific performances, evolving through interaction and setting [10–12]. Stories from other new parents could be interesting, practically useful, relevant and credible, while passing empowering messages about lay knowledge and self-efficacy [10–12, 34]. Storytelling formed part of a rite-of-passage ritual, initiating women into a community of mothers with emotional support and concrete, practical information [12, 17, 34].

Birth stories may reinforce normative values, appropriate behaviour or institutional compliance [3, 10, 11, 34]. Meanwhile, some personal accounts disrupted dominant, oppositional narratives, including medical/natural discourses, by encompassing contradictions and presenting passivity and action, empowerment and subjugation, or antagonism and harmony [10, 11, 33].

These findings regarding birth stories recall research and theories on storytelling more generally. Stories offer a sense of authenticity, flexibility to personal interpretations, multiplicity of messages, resonance with other stories and information, ability to navigate complexity, openness to uncertainty, and community-building capacity [31, 35, 36]. Compared to didactic teaching methods, birth stories – as grounded and interactive, yet wide-ranging, performances – may provide a variety of advantages and possibilities for pregnant and birthing women.

## Methods

### Aim

Through participant observation, the pilot study sought to understand how group-led antenatal sessions co-produced birth knowledge. Specifically, the study asked what types of knowledge emerged from antenatal sessions, how participants navigated different birth knowledges, and what was the role of storytelling. This paper focuses on this final question: How did birth stories contribute to the co-construction of birth knowledge in antenatal sessions?

### Design

The nuances and multiple understandings of birth and birth knowledge require a methodological and analytical framework that enables complex readings grounded in everyday experience. As a learning process committed to insider-knowledge [37], participant observation mirrors the knowledge development practices under investigation.

Coding and analysis proceeded according to template analysis, a flexible form of thematic content analysis [38], and narrative analysis. Dialogic narrative analysis engages multiple, contradictory meanings and overlapping contexts [31, 39]. Beyond verbal content, clues about the meaning and ‘work’ performed by stories emerge from devices like character traiting, agency, silence, resonance of other stories and context, such as interaction with the audience, locality and wider socio-economic structures [31]. This theoretical, methodological and analytical stance recognises the co-construction of meaning, participants and wider contexts that lies at the centre of this inquiry.

Feminist ethnography also shaped the research design and process. Principles include a commitment to reduce exploitation and support positive change [37, 40], acknowledgement of the complex subjectivity and reflexivity of researcher and participants [40–42], and a recognition of authentic truths as co-produced, incomplete, contingent and changeable [41–43]. As a pregnant mother learning about the experiences of childbearing women, the researcher developed an awareness of conundrums around body knowledge [44], feminist solidarity [42], and insider research [41, 42].

### Participants

Using purposive sampling, the research incorporated 43 attendees of Homebirth and NCT antenatal sessions. While these two data sources were not comparable in format or size, the analysis occasionally looks to similarities and differences to contextualise general findings, suggest certain patterns and propose areas for future research.

Homebirth sessions formed the focus of analysis, as these sessions best matched the subjects of study: the event of childbirth and mother-to-mother knowledge sharing practices. The researcher audio-recorded and transcribed three sessions over a total of 6 hours, incorporating 25 participants (seven men and 18 women). Of the Homebirth women, 10 were pregnant, including four for the first time and eight for a subsequent time (including the researcher responding to direct questions), and eight were postnatal and non-pregnant (including two facilitators and two doulas). Different people attended each session, apart from the researcher and one facilitator who attended twice.

A series of teacher-led NCT classes on pregnancy, birth and postnatal life provided another type of data. The researcher hand-recorded and transcribed six sessions over a total of 13 h, incorporating 18 participants (eight men and 10 women). All of the NCT women were pregnant for the first time, except the teacher and a postnatal visitor from a previous class; the researcher did not participate in these sessions. The same people attended each session, apart from sickness absence and a postnatal couple who attended part of one session.

Demographic information was not collected, however all sessions took place in mixed-demographic suburban areas. Participants appeared disproportionately white and middle-class compared to the local neighbourhood.

### Data collection and setting

At each Homebirth session, the researcher audio-recorded and took notes on nonverbal information while sitting in the circle with other participants. These free, drop-in sessions took place on weekend mornings in a private room in a community space, and included some participants’ children. Each meeting comprised introductions, an invitation by the facilitator for one or more postnatal women to tell a birth story, and open questions and discussion.

For the NCT group, the researcher hand-recorded proceedings from the corner of the room, while other participants sat in a partial circle facing the teacher. These pre-booked, fee-paying sessions took place on weekday evenings in a church. Classes primarily took the form of lessons, with some discussion, demonstration and team-building exercises.

In all cases the researcher transcribed recordings and observations within1 week, removing all identifying details and replacing names with pseudonyms. Basic annotations regarding expression and nonverbal content aimed to strike a balance between readability and exact representation.

### Data analysis

Template analysis helped to organise the data and begin analysis. This flexible process enables examination of the significance of and relationships between themes, without losing sight of context and individual voices [38]. Template analysis requires the researcher to develop a ‘template’ of key themes by reading and re-reading data [38]. The template provides a framework for more in-depth analysis, in this case using elements of narrative analysis and feminist ethnography.

The researcher began with the first Homebirth transcripts, marking excerpts with one or more themes as applicable. She focused on themes relating to the research questions but remained open to other themes as well. The next two Homebirth transcripts suggested a few more themes, and the NCT transcripts offered many more themes. She then re-read all transcripts at least once, marking and re-marking excerpts while adding, discarding, consolidating and rearranging themes and subthemes until she achieved a clear, concise and comprehensive template. The final template included three groups of themes: topics of discussion (e.g. self, midwives, labour techniques), types of knowledge (e.g., authoritative, intuition, personal experience) and techniques for navigating knowledge (e.g., comparison, humour, story). Variations on control (control, compromise and chaos) acted as integrative subthemes running through the main thematic groups.

Following construction of the template, the researcher considered the prevalence of themes and combinations of themes. This initial analysis indicated patterns in the data worthy of further exploration. For example, humour often associated with topics of extreme concern, and topics of self-experience appeared most frequently in stories. The researcher then sought to understand patterns and correlations by using elements of narrative analysis and feminist ethnography (as described above) to explore particular excerpts with the broader context of the data. This gradual process enabled the researcher to draw key themes and patterns clearly from the data.

### Ethical approval

A university Research Ethics Committee approved the project in May 2016. Before observations, the researcher sought initial approval from gatekeepers, identified support systems in case of participant distress, and made available a participant information sheet (PIS) in advance. At the start of the first NCT class and each Homebirth session, the researcher distributed paper copies of the PIS and consent forms. She sought consent only after verbally introducing the project, giving everyone a chance to ask questions, and offering to leave if anyone felt uncertain.

## Results

Storytelling proved central to mother-to-mother antenatal group practices, providing not only information, but also a means for understanding. Analysis of antenatal session transcripts suggested that birth-related storytelling facilitated learning through various mechanisms: Stories (re)shaped expectations, shared practical techniques, navigated different truth claims and approaches to knowledge, and helped build supportive communities of parents. These findings emerged more prominently in group-led sessions compared to teacher-led sessions.

### (Re) shaping expectations

Women in Homebirth sessions often used stories to answer questions about childbirth, and shape and reshape expectations about labour. Contrasting tales revealed a wide variation in labour experiences, for example in a Homebirth discussion where four women discussed ‘pushing’ during the second stage of labour. Using several first-hand and second-hand stories, the excerpt demonstrated some consensus (“you just have to listen to your body”) alongside contrasting bodily sensations (“your body will push,” “I felt like I did push,” “they did not push at all, they just breathe”), labour experiences (a rapid second stage, or a long delay between second-stage contractions) and interactions with midwives (“telling you to push against a closed door,” saying “just wait”). In addition to presenting a range of expectations for childbirth, this excerpt depicted a range of approaches to knowing childbirth, for example trust and resistance to intuition, and positively and negatively ceding control to health professionals. This aspect of storytelling bears further discussion below.

Either in relation to direct questions or more spontaneously, stories also reported emotional and physical self-experiences that rarely appeared in teacher-led discussions. Subjective accounts vividly related the embodied experience of childbirth. For example, Emma described her sensations in detail during the second stage: “But the feeling of him actually crowning, and then, the relief when that happened. And then the feeling of his shoulders rippling for the next one, I will never forget that. Ripple. <Laughs>.” In another excerpt, several women discussed their experiences of pain:

*Excerpt 1*

Holly: I think it is important to trust your instincts as well, because I knew when I was fully dilated and she was coming. And I told the midwife, and she was like, oh no, if you were in that final stage you’d be in so much more pain, you wouldn’t be able to cope with it. <Laughs> …I remember them asking, on a scale of 1 to 10, how much pain are you in? And I wasn’t thinking about that. But I remember people saying you have to lay it on thick. So like, oooh, 8, 9! <Laughter>.

Becky: It’s not always pain is it, it’s a sensation. My husband kept asking me, <gruff voice> are they strong, like? How painful, are they painful? And I was just like, I don’t know. I just carried on doing my thing.

Sarah: People say, other friends that have babies. I dunno, it’s not pain, like pain-pain –.

Becky: Like stubbing your toe, or breaking your leg.

Sarah: Yeah, for me, it was something completely different. And manageable.

Holly: Because I didn’t know before I had Jack that it wasn’t just going to be constant.

Sarah: Yeah, when it’s strong you can just think, in a minute, it’s gonna pass. Keep breathing. And I think as well from TV, and films and things, you think it builds to this crescendo. But for me, the worst bit was the first stage. And when it got to the breathing baby down stage, or pushing, or however you want to describe it, like, that was a relief.

Nicola: Yeah, I agree, yeah.

This exchange challenged mainstream equivalences between childbirth and pain, while affirming the intensity of subjective experience and the role of intuition. The women complicated typical portrayals of childbirth pain by using humour, refusing to quantify or externalise their experience, and emphasising the existence of other physical sensations. Like many examples of storytelling, this discussion worked on several levels, not only shaping childbirth expectations, but also sharing practical advice, and working to build a positive homebirth community. These mechanisms receive further discussion below.

A further Homebirth excerpt regarding placenta delivery provided an example of women discussing an oft-overlooked aspect of childbirth. Beginning with the consensus that many women had felt unprepared for the third stage, the conversation turned to issues around receiving the syntocinon or ergometrine injection to facilitate placenta delivery. Several women told personal stories that illuminated a range of experiences of the third stage (placenta coming naturally after an hour, or 9 hours) and of decision-making with midwives (receiving the injection without being asked, being told the injection was needed after 20 min, negotiating about the injection with regard to other factors like bleeding). As in stories about pushing and the physical sensations of childbirth, multiple stories about the final stage of labour highlighted new issues for pregnant women to consider, and enabled diverse expectations and interpretations.

### Practical guidance

Situated in real-life contexts and often presented in an engaging manner, stories effectively passed on numerous practical techniques and ideas for birthing women. Due to the dearth of first-hand childbirth experiences, NCT participants shared relatively few stories. However the following exchange of second-hand stories addressed the issue of postnatal perineal soreness:

*Excerpt 2*

Lauren: My friend also said she was sore down below, and she was surprised by that.

Megan: Apparently you have to pour a glass of water when you wee because it burns…

Shelley: And the first poo –.

Sophie: It’s really traumatic!

Diana: My friend said it was like having a baby again and in the end she just had to stand up, she didn’t care. It was so awful.

Here, anecdotes enabled discussion about a key topic of concern – genital damage – that appeared fleetingly but repeatedly in the NCT group, usually framed with humour, stilted speech and/or silence. Corroborating anecdotes helped these women to acknowledge mutual fears and share specific ideas that could help.

Homebirth stories frequently detailed descriptions of how women prepared for childbirth, or dealt with unexpected events during labour:

*Excerpt 3*

Emma: One thing that helped me was to have my in-case-of-hospital plan written down.

Alex: In case of hospital I’ll be like, general anaesthetic, sedate the hell out of me. I’m just so phobic of hospitals, I’m scared of doctors touching me…

Emma: I had a list of things I’m willing to accept, things that are absolute no go – <Alex: Yeah – > and things that if you prove to me this is a genuine medical emergency I’ll take it on – <Alex: Yeah – > and that my midwife knew it word for word. We submitted it to the hospital and made sure they knew it word for word – <Alex: Oh, really – > I could relax into it then…

Sarah: Can you say, if I go to hospital, can I only see a midwife?

Alex: Oh yeah, true. One of my friends had her baby at 35 weeks interestingly. She says, her memory of birth is like, sitting in a pool, breathing away, her husband just in the corner quietly. He says that’s not true, I was outside signing every disclaimer under the sun ‘cuz you were 35 weeks and they wanted to intervene and they wanted to do this and they wanted to do that. And he was like, I spent your entire labour arguing with people, to get them to leave you alone. So, I could just do that.

Beginning with humour and escapism (“sedate me”), these anecdotes compiled practical suggestions (“a list,” trusted midwife, partner as mediator) on the crucial issue of how women may feel secure amid the inherent unpredictability of labour. In another excerpt, stories provided further, sometimes contrasting ideas for dealing with change (writing a birth plan, refusing to write a birth plan, packing a just-in-case hospital box, maintaining open dialogue with carers). Beyond the ideas it contained, a personal account acted as a practical example in itself, of a woman who passed through the uncertainty of labour and emerged to tell the story.

Homebirth discussions and stories often highlighted practical methods for facilitating and managing advanced labour. For example, Louise gave a detailed rundown of the use and success of various techniques during her labour (namely, a bath, TENS machine, birthing pool, music, candles, breathing, hypnobirthing, gas and air). Emma’s story described her active birth methods, including “trying to crab-walk sideways up the stairs” to help labour progress, and changing position to adjust baby’s angle of descent.

Several tales indicated how to deal with other people during labour, like Becky’s cautionary tale about her partner (“My husband was kind of stroking my arm and whispering sweet nothings… But what I wanted was to say, it’s okay, I’m fine, it’s a bit distracting”) and Holly’s account of managing her caregivers:

*Excerpt 4*

Holly: So I basically locked myself in [the hospital bathroom] on my own because I just didn’t want anyone around me. And when I went to the pool, I was just really bossy with the midwives. I was like, there’s a wall there, and I was like, <mock shouting> go and stand behind that wall! I don’t want to see you! <Laughter. > And there was nowhere else for her to go really. <Laughs. > She just kind of stood behind the wall and I could see her little clipboard. Like, <mock shouting, pointing> I don’t even want to see your clipboard! <Laughter. > So I just didn’t want people around me.

This entertaining anecdote – made funnier by the soft-spoken manner of its teller – fleshed out the notion that birthing women often seek privacy. By presenting practical tips in an individual context, stories offered more useful information on how to use various techniques or ideas.

### Navigating knowledge

In addition to proposing practical techniques for labour, some stories suggested ways to negotiate different truths around childbirth. Stories often acted as mediators, bringing together complex arrangements of authoritative or quantitative knowledge, intuition or chaos. For example, Becky’s and Sarah’s exchange of stories about labour progression placed quantitative knowledge (centimetres of dilation) within the context of embodied experiences (pain or disappointment as a result of vaginal examination) and other physical signals (the labouring woman’s position, behaviour or vocalisations).

In another example, a story provided the focal point for a Homebirth discussion about due dates to strongly disparage authoritative claims as manipulative and inadequate sources of knowledge:

*Excerpt 5*

Laura: They say the placenta can –.

Debbie: Calcify – <Laura: Yeah – > But calcification doesn’t have any impact on the placenta, it happens anyway.

Steph: But there are so many different options aren’t there, if it pleases them you can opt for the monitoring.

Joanna: And it’s not the nicest thing to talk about and they don’t tell you but it can happen anytime. So actually the monitoring is better…

Laura: It’s quite scary isn’t it, because if it’s anything to put your baby at risk, and then it’s that whole fear because obviously you don’t want to put your baby at risk…

Debbie: At the conferences yesterday. There was a speaker who had two homebirths, they lost their middle daughter. But she said, with her first I think it was, she was overdue and she didn’t want to be induced. And this consultant sat down at the edge of the bed and said, statistics, you’re putting your baby at risk. And the question she asked, and I think it’s an amazing question to ask, was… what is the risk to me, right now, and this baby? And the consultant was like, you’re not supposed to ask questions! You’re just supposed to go, oh no, that’s terrible risk! … The second time for them, it was a really massively rare thing. There are no statistics for that happening… It’s not even a nought point 1 % statistic thing. And there was nothing anybody could have done about it… And you’ve taught two whole generations of women, everything will be fine for 42 weeks and then your baby goes off! <Assent>.

Steph: Flips a little switch, that’s it… They should do what they do on the continent. Like, you’ll probably have it in, October. <Laughs.>.

This excerpt mitigated external guidelines about due dates with humour, comparison (“what they do on the continent”), and alternative techniques for evaluating safety post-dates (e.g., foetal monitoring, consideration of other aspects of the individual’s situation). Perhaps most strikingly, the story discredited authoritative knowledge (“statistics”) by introducing chaos – the uncontrollable unknown – with reference to the unexpected tragedy of stillbirth.

A story could also recount how women made decisions during labour, a sort of knowledge-in-action, for example using the BRAIN decision-making technique (Benefits, Risks, Alternatives, Intuition, Nothing). While formal presentations of the BRAIN method in NCT and Homebirth groups were fairly abstract, Louise’s account described how the method worked in practice:

*Excerpt 6*

Louise: When the midwife came, she said my blood pressure was high. So when the next midwife came along, she said, oh it’s still high and we’d like to transfer you to hospital. So Dave put into use what we learned on the hypnobirthing course saying, you know, is the baby’s heart rate okay? Yes, that’s fine. Well, what are the risks? There is a small risk she could have a seizure. Um, okay what are our options? And they said well, we are happy to just monitor and as long as it stays the same, it was a go. It was a bit of a pain, when I was in the pool they had to keep taking my blood pressure and things… Then, just when he was like really nearly there, um, they took my blood pressure and it was really high. And so they said, we’re going to have to make the call to call an ambulance. Um, but, they said, it takes like 20 minutes for the ambulance to get here, and he’s really nearly there. So basically just crack on. <Laughter. > Ah, so I did. And he shot out <laughs> across the pool.

This empirical example turns abstract, medicalised notions of risk into a concrete, understandable set of concerns. In addition to advocating specific questions to facilitate decision-making, the excerpt portrays lay people – Louise and her partner – as suitable decision-makers. Further, this story shows the role that midwives played in negotiating risk, emphasising a collaborative approach to decision-making and knowledge. Like other practical techniques for labour, story-based demonstrations of decision-making during childbirth offer specific, grounded examples from which women can learn.

### Building communities

At NCT and Homebirth groups, stories displayed a capacity to build communities by fostering positive, empathetic communication between parents. In many cases women appeared to draw confidence and support from story-based interactions during the session. This effect corresponded with both organisations’ central aims to increase parental confidence [45, 46].

Further, especially in the case of the Homebirth group, stories built positive visions of physiological childbirth behind which women could unite. Although neither group mentions this aim in their core statement of purpose, the message seems implicit: Most medical interventions are unavailable at home, and the NCT retains links to its historical emphasis on ‘natural’ birth [47]. This awareness of the groups’ agendas recalls Cosslett’s recognition that a peer group may provide assistance, discipline, or both [10].

For example, many Homebirth discussions prioritised non-medical approaches (e.g., physiological third stage, non-medical pain management), or associated hospital with disrespect, trauma or negativity (e.g., Excerpt 3, Emma’s references to her first birth, Laura’s story). Some anecdotes advocated physiological birth by minimising the role of pain (e.g., Excerpt 1), bypassing negative elements with humour (e.g., Joanna on perineal stitches and in Excerpt 8), or envisaging birth as pleasurable:

*Excerpt 7*

Sarah: My first birth was lovely, because when she was born at 5:40 in the morning, at the end of June. So it was like midsummer and the sun was coming up. And I could see out my doors into the garden, and the first morning light was the only light. So I was just staring, it was really peaceful and calm and lovely. I will always remember that moment.

While some women might have found Sarah’s description alienating or unrealistic, to others it could offer a reassuring, positive perspective rarely encountered in medical or mainstream representations of childbirth. This latter response seemed palpable at the group, compounded perhaps by the face-to-face context, and Sarah’s inclusive, accessible manner.

Alternative pictures also appeared at the Homebirth groups. Sarah recalled women who chose to birth in hospital, and Holly described her first birth as “a really lovely experience even though it was hospital. I was in the pool and it was really positive.” Further, by recognising and contextualising negative elements, stories may reassure women about their ability to face all eventualities:

*Excerpt 8*

Joanna: And then all of a sudden, like you were saying before, it just went from nought to 60 and I was like, oh my. And I was pulling at my hair, I was thinking, I didn’t even think this was transition. I was, I thought, <rapid voice, rising pitch> what is going on, I am not coping, I didn’t have pain relief last time, oh there’s no water in my pool, can’t even get in me pool. So I tried to get my TENS machine on, I had my phone taking pictures, they were all skew-wiff. So I just whacked it up to max and I were like, <mock panic> it’s not working!... I was saying, I can’t cope, I can’t cope. And Si was like, oh, these seem really intense these, love. I was like, yeah, fine, just tell me I’m being silly.

Joanna’s comedic tone, in the face of apparent pain, desperation and self-doubt, evoked much laughter, perhaps helping women build resilience amid the uncertainty of childbirth. Such confidence- and community-building work became apparent at several moments during the groups, such as the spontaneous clapping following Emma’s birth story. Even the story-based NCT exchange about perineal tears, by overcoming silence and taboo, engendered a feeling of community around shared fears and afflictions (Excerpt 2). Stories provided a vehicle for people to reassure one another, find common ground, and express solidarity, drawing upon and contributing to a supportive community of parents that may transcend any single antenatal session.

While participant observation could not establish the impact of storytelling within antenatal sessions, the content and context of stories suggest manifold capacities. As described above, stories worked to (re)shape expectations, share practical guidance, navigate birth knowledges and build communities. Reflection on these findings from the perspective of wider literature offers some ideas about the effects of birth stories on childbearing women.

## Discussion

Like stories in general, birth stories told in antenatal sessions performed educational work in a number of ways as described above. While the capacities of storytelling in antenatal sessions will benefit from further investigation, these findings have implications for practice in childbirth education, parental support and maternity care generally.

Returning to Frank’s notion of narrative ambush [31], the face-to-face, personal accounts told in antenatal sessions seemed to possess a capacity to shape and reshape expectations of childbirth. While listeners undoubtedly found some anecdotes resonated more than others, the range and depth of expression often seemed to effectively engage listeners. Social context, vulnerability of teller and tale, and shared emotion all contributed to this process. Further, many stories went beyond simple delineation into positive (normal, everyday, easy) or negative (dangerous, risky, medicalised) to contain moments of both and beyond.

In passing on practical techniques, stories did more than provide abstract instruction. For example, Holly expressed her desire for privacy (Excerpt 4) arguably more usefully, and certainly more humorously, than Michel Odent’s essentialising imperative to solitude because “the mammalian needs must be met” ([48], p. viii). Story-based examples did not require women to turn into mammals or disembodied uteri – the birthing women in stories retained their fears, hopes, preferences and identities.

Storytelling, alongside other forms of mother-to-mother engagement in antenatal sessions, played a special, augmentative role in navigating birth knowledge. By weaving together multiple approaches, stories seemed to increase the agility and effectiveness of other types of knowledge. For example, Joanna’s reference to the pre-defined notion of ‘transition,’ followed by an embodied account of her experience (Excerpt 8) formed a particularly convincing description of that phase of labour. In this case, authoritative knowledge and subjective experience together formed a more credible truth claim than either in isolation.

Stories increased participants’ access to types and techniques of knowledge that are underrepresented in a classroom setting, such as intuition, subjective experience or comparison. In part, the relative absence of stories in the NCT classes must have related to the dearth of first-hand childbirth experience in the room. Scholars have also suggested that society perceives subjective, embodied and internal knowledge as lower status than authoritative, evidence-based or externally-observable knowledge [49–51]. The NCT for example, draws heavily on associations with scientific evidence to build its identity, educational programmes and campaign messages [47, 52]. Nonetheless, these less ‘teachable’ approaches to knowledge, and the combination of multiple approaches, enabled Homebirth women to address a multitude of issues around childbirth with depth and nuance.

Flowing between different types of knowledge to reinforce, challenge or contextualise different claims, stories often showed a dialogic relationship between truths. In addition, stories about decision-making in labour showed how a multifaceted, collaborative approach could work in reality. Storytelling in antenatal sessions acted as a form of situated knowledge [28], like other feminist constructions of credible knowledge as complex, fluid and embedded in context (e.g., [25, 26, 50]). Through specific, grounded examples, stories meaningfully displayed a range of approaches to knowledge rather than a single route to truth.

Finally, the community-building capacity of birth stories often reflected and reinforced its other work, shaping expectations, passing on practical information, and helping women to navigate multiple truths around childbirth. A social context increased the capacity for stories to engage listeners on many levels. Connections facilitated by storytelling may contribute to personal relationships, extending this community-building work beyond the session itself.

### Limitations

This study was primarily limited by the lack of availability of group-led sessions, despite NICE recommendations for participant-led antenatal education [16]. The only group-led sessions accessible to the researcher within the timescale focused on homebirth, and took place in relatively middle-class suburban areas. A group-led session for refugee and asylum seeker women was unavailable due to limited research resources and the level of English required for reading consent materials. As for teacher-led sessions, potentially more demographically diverse National Health Service (NHS) classes were unavailable due to time constraints. Given these circumstances, participants disproportionately represented a white British, middle-class demographic. Additionally, many Homebirth participants focused on physiological birth and already seemed to possess a basic knowledge of childbirth.

The research methodology contained its own limitations. While participant observation offers certain insights that may not have been visible in interviews or questionnaires, it can also mask dissent and overlook taboo topics. The researcher used wider literature and previous experiences to identify absences in the data and infer meaning, for example whether silence on a topic signified disinterest, agreement, disagreement, or acute concern.

Finally, the researcher was in many ways an ‘insider’ in these groups, as a white, middle-class – and for some of the time, pregnant – mother. As the researcher’s position – like any researcher position, ‘insider’ or otherwise – bears some relevance to the findings, it warrants brief exposition here. Personal immersion in the subject provided a valuable opportunity and resource, for example as the researcher’s pregnancy notably prompted a more engaging, open response from participants. Further, the researcher could access her own embodied knowledge and experiences in understanding participants’ meanings. Nonetheless the researcher recognised her perspective as partial and limited by her personal experiences, demographic position and other circumstances. In analysing the data, the researcher kept participant voices at the forefront, and recognised her own perspective as one among many influences on the project, namely the countless contributors to articles and books on historical and contemporary childbearing experience, and hundreds of informal conversations with other parents over a decade of motherhood.

### Future research

The next phase of research seeks to expand the scope of the pilot study. Alongside participant observation, research data will draw upon archival research, questionnaires, postnatal focus groups, and interviews with childbirth educators. In addition, the researcher will engage with NHS antenatal sessions and non-homebirth focused group-led sessions. With this mixed methodological approach, a broader demographic, and a wider range of perspectives on childbirth, the researcher will further explore pilot study findings, investigate informal collective practices, and seek to evaluate the impact of collective knowledge on childbirth experiences.

Other researchers may seek to attend to the role of birth stories and collective learning in other geographical or historical contexts, or to quantitatively evaluate the impact of a particular intervention. Above all, research that seeks to improve pregnancy and maternity experiences must reflect the complex and diverse needs, preferences and realities of birthing women.

## Conclusions

Teaching the physiological mechanics of birth or procedural details of hospital policy is not enough. Effective antenatal preparation should improve women’s abilities to navigate childbirth discourses and realities by engaging multiple approaches to knowledge. Storytelling is a type of collective birth knowledge that provides additional resources for childbearing women by shaping expectations, sharing practical techniques, mediating between different types of knowledge, and building communities. As dialogic, complex and flexible learning tools, birth stories told within antenatal sessions offer uniquely diverse, credible and supportive messages.

Birth stories are not a specialist intervention, but rather an educational resource available to anyone who has ever had or heard about a childbirth experience. Unsurprisingly, Homebirth sessions that incorporated pregnant and previously-delivered women drew on a greater wealth of experience than the NCT group of first-time mothers. A mixed cohort enabled more storytelling, and more representative entanglements of childbirth knowledge.

By exploring how storytelling operated in antenatal sessions, this paper offers a number of implications for practice by childbirth educators, midwives, other medical practitioners and health authorities. Firstly, this paper elaborates unheeded NICE guidance for improving antenatal education provision for women and partners by offering participant-led sessions [16]. These findings add to a growing body of evidence advocating antenatal sessions that include a mixed cohort of first-time mothers and previously-delivered women, also seen in the acclaimed Albany Midwifery Practice [17], successful antenatal interventions for asylum-seeker and refugee women [53], and a new model for group antenatal care [54]. Meanwhile, previous studies have suggested that women need more access to ‘positive stories’ (e.g., [1, 29, 30]), and optimistic, flexible, realistic portrayals of childbirth (e.g., [9, 55]). These findings reiterate that a range of in-depth birth stories, told face-to-face in a supportive context, may offer learning opportunities with the capacity to improve childbirth experiences. Health authorities and childbirth educators need only facilitate and normalise this practice of sharing birth stories.

## Data Availability

The datasets used and/or analysed during the current study are available from the corresponding author on reasonable request.

## Abbreviations

NCT: National Childbirth Trust
NHS: National Health Service
PIS: Participant Information Sheet
